# Development and implementation of Phleos, a web-based tool for the data collection on Hypereosinophilic syndrome: the Italian Network on HES (INHES) study protocol

**DOI:** 10.3389/fimmu.2025.1638798

**Published:** 2025-07-16

**Authors:** Stefania Nicola, Marco Caminati, Richard Borrelli, Luca Lo Sardo, Federica Corradi, Iuliana Badiu, Angelo Vacca, Palma Carlucci, Amato De Paulis, Ilaria Mormile, Marco Zurlo, Mario Di Gioacchino, Vincenzo Patella, Stefano Del Giacco, Giulia Costanzo, Simone Negrini, Giovanni Rolla, Luisa Brussino

**Affiliations:** ^1^ Immunology and Allergy Unit - AO Ordine Mauriziano di Torino, Turin, Italy; ^2^ Department of Medical Sciences, University of Turin, Turin, Italy; ^3^ Department of Medicine, University of Verona, Verona, Italy; ^4^ Allergy Unit and Asthma Center, Verona Integrated University Hospital, Verona, Italy; ^5^ Internal Medicine Guido Baccelli, University of Bari Aldo Moro Medical School, Bari, Italy; ^6^ Department of Translational Medical Sciences, Federico II University, Naples, Italy; ^7^ Center for Basic and Clinical Immunology Research (CISI), WAO Center of Excellence, University of Naples Federico II, Naples, Italy; ^8^ Institute for Clinical Immunotherapy and Advanced Biological Treatments, Pescara, Italy; ^9^ Department of Internal Medicine asl Salerno, “Santa maria della Speranza” Hospital, Salerno, Italy; ^10^ Postgraduate Program in Allergy and Clinical Immunology, University of Naples Federico II, Naples, Italy; ^11^ Department of Medical Science and Public Health, University of Cagliari, Monserrato, Italy

**Keywords:** hypereosinophilic syndrome (HES), HES, eosinophils, Phleos, eosinophilic-related disorders, web-based tool, INHES

## Abstract

Hypereosinophilic syndrome (HES) is a heterogeneous group of disorders characterised by persistent hypereosinophilia associated with organ damage. Due to its rarity and heterogeneity in its clinical presentation, HES remains underdiagnosed or misdiagnosed, often leading to delayed diagnosis and irreversible organ damage. The complexity of HES diagnosis is even more complicated due to the absence of standardised criteria. Moreover, the lack of structured referral pathways among specialists, including allergists, clinical immunologists, haematologists, and rheumatologists, further hinders optimal patient care. To address these challenges, the Italian National Hypereosinophilic Syndrome (INHES) Network aimed to enhance the diagnosis, management, and research of HES. INHES objectives also include connecting specialised centres, facilitating data collection on HES and eosinophilic-associated conditions, and improving healthcare standards through consensus guidelines. To do this, INHES has created a web-based platform called “Phleos” to develop a comprehensive referral map, ensuring timely and appropriate patient access to expert care. Phleos is a web-based datasheet, per GDPR regulations, to systematically collect anonymised clinical data, including absolute eosinophil counts, organ involvement, laboratory parameters, instrumental assessments, and treatments. A structured classification system enables differentiation among idiopathic, lymphocytic, myeloid, familial, reactive, and overlap forms of HES. Moreover, the platform integrates standardised diagnostic pathways and treatment protocols to harmonise patient care across participating centres. The INHES Network aims to mitigate diagnostic delays, optimise therapeutic decision-making, and advance research in eosinophilic disorders by fostering interdisciplinary collaboration and establishing a unified framework. This initiative represents a crucial step toward a cohesive national strategy, ultimately improving clinical outcomes for HES patients in Italy.

## Introduction

Hypereosinophilic syndrome (HES) defines a heterogeneous group of different conditions within the wider umbrella of hypereosinophilic disorders, characterised by blood hypereosinophilia and related organ/systemic damage (1).

Similarly to other rare diseases, the recognition and management of HES and its subtypes is hampered by a number of practical challenges, from the variability of the available definition of the disease to the limited experience commonly characterising non-specialised physicians, potentially leading to misdiagnosis. In fact, on one side, overestimation may occur if diagnosis relies on increased blood eosinophils only, whether or not the cut-off > 1500 cells per microliter is verified, without damage demonstration (1, 2). On the other side, HES heterogeneous clinical features, together with the absence of specific, unequivocal findings on physical exams, lab tests and imaging, might result in underdiagnosis or misdiagnosis in the context of eosinophilic dysimmune conditions.

As a consequence, the correct identification of the disease may occur after a long time from its onset when organ damage with poor reversibility has already developed (3).

In addition, the referral to specialised centres/physicians also represents a challenge; in fact, a variety of diverse specialists take the lead in HES management, including allergist/clinical immunologist, haematologist, internal medicine doctors, pulmonologist, rheumatologist, according to the peculiarities of the setting where they work (2, 3).

However, if multidisciplinary management is nowadays univocally considered the best approach, a substantial lack of standardised protocol in terms of treatment priorities, follow-up timing, and organ assessment can be observed even among the referral centres.

In the field of rare diseases, the positive impact of registries on network implementation and overall physicians’ awareness has been described (4, 5).

The present report aims to describe the goals and methods of the first Italian collaborative national Network on HES (INHES—Italian Network on HES) for patients and physicians.

Patients will take advantage of an updated and, as much as possible, complete overview of referral centres for HES distribution across the Country. From the clinicians’ perspective, a structured network will facilitate data collection and sharing as well as the development of a “common language” in terms of disease management.

### Objective

The specific objectives of the collaboration project are the following:

- Connecting Italian centres with expertise on HES across the whole Country
- Collecting data on HES and eosinophilic dysimmune conditions for clinical and scientific purposes
- Enhancing the standard of care for HES patients in referral centers by codifying and spreading common recommendations for the management of the disease to prevent irreversible damage definitely.
- Increasing the overall knowledge of HES at different levels of the Healthcare System
- Providing a national map of centres with specific expertise and facilitating the referral

### Ethics

The study was approved by the local ethics committee (Comitato Etico Territoriale Interaziendale (CET) “A.O.U. Città della Salute e della Scienza di Torino”, protocol code 61.034.049), and will be wholly conducted following the Declaration of Helsinki and the latest Best Clinical Practice guidelines. All the enrolled patients will release their informed consent for publication. The consent will be securely stored and available, if requested.

## Materials and equipment

### Data storage

The platform named “Phleos” will be engineered using advanced cloud technologies, employing a secure database system. The platform will be created for the specific purpose of entering and managing anonymised data from patients. The platform will be built in compliance with all the rigors related to the European law on the protection of personal and sensitive data GDPR 2016/679.

Data will be entered by clinicians or healthcare professionals managing eosinophilic-related disorders. The platform will be accessible only to selected users, following registration and approval of the username by the scientific board. All collected data will remain editable, allowing for the correction of any errors or the addition of missing information.

All data entered will always be accessible to the users who entered it. The entire dataset will be available, if requested, upon approval by all participating centers.

## Methods

### Study population and sample size

All the patients with eosinophilic-associated disorders will be included in the study and potentially in the online platform.

Due to the prospective nature of the study and its aim, no sample limits are set at the moment.

### Participants and general information

Participants will be anonymised with a unique subject ID and pseudonym, and their data will be anonymously stored in an appropriately secured server.

Data concerning the gender of the patient, the actual age and the ages of disease onset and diagnosis will be fulfilled.

The following sections refer to the patient’s living conditions and home treatments, including steroids. If the patient is taking steroids at home, the maximum levels of eosinophils on-treatment and off-treatment should be noted.

If the patient is dead, the user should describe the time and causes of death.

### Levels of absolute eosinophils count

#### Peripheral blood eosinophil count less than 500 cell/mcl

If a patient has less than 500 EOS/mcl with evidence of any targeted organ damage, the user will choose the organ that has been involved. Moreover, a final diagnosis linked to the damaged organ can be selected. **Table 1** shows the list of potentially involved organs and the eosinophil-related diagnosis.

**Table 1:** Potentially involved organs and the eosinophil-related diagnosis.

Targeted organs	Eosinophil-related disorders
Oesophagus	Eosinophilic esophagitis (EoE), other
Duodenus	Eosinophilic enteritis, other
Stomach	Eosinophilic gastritis, eosinophilic gastritis, and other
Colon	Eosinophilic colitis, other
Lung	Acute eosinophilic pneumonia, chronic eosinophilic pneumonia, eosinophilic bronchitis, Allergic BronchoPulmonary Aspergillosis (ABPA), eosinophilic asthma, other
Kidney	Eosinophilic cystitis, eosinophilic prostatitis
Skin	Eosinophilic panniculitis, eosinophilic cellulitis, necrotising skin, eosinophilic vasculitis, eosinophilic fasciitis, eosinophilic folliculitis, eosinophilic mouth ulcers.
Heart	Eosinophilic myocarditis, eosinophilic pericarditis
Muscular system	Eosinophilia-myalgia syndrome, other
Ear, nose, and throat system	CRSwNP/CRSsNP, allergic rhinitis, NARES, and other
Vascular and haematological system Central Nervous System Peripheral Nervous System Eye Other	Free text field

#### Peripheral blood eosinophil count between 500 and 1500 cell/mcl

If the patient shows AEC between 500 and 1500 cell/mcl, the user should select three different options on how the patient was assessed regarding organ damage detection:

1. As the HES criteria are not met, no further assessments were done
2. HES criteria are not met, but due to the high risk of HES development, a watch-and-wait approach was taken
3. The patient was considered as affected by HES, so diagnostic and therapeutic pathways were taken

After that, the user can select one or more underlying causes responsible for the eosinophilia.

The following conditions are listed: ABPA, EGPA, Otitis media, Sarcoidosis, Atopic dermatitis, Hashimoto’s thyroiditis, Haemolytic anaemia, GVHD following HSCT, Liver or biliary disorders, Serositis, Immunodeficiency, Asthma, Bullous skin disorders, GVHD, Gleich Syndrome (cyclic angioedema with eosinophilia), Connective tissue disorders, Dermatitis herpetiformis, Inflammatory Bowel Disease (IBD), Allergic Rhinitis, Reactive HES (drugs, DRESS, infections…), CRSwNP/CRSsNP, other.

#### Peripheral blood eosinophil count over 1500 cell/mcl

In the case of a patient showing hypereosinophilia, the first step was to discriminate between the different forms of HES or HE: lymphocytic HES (L-HES), myeloid HES (M-HES), familial HES, overlap forms of HES, idiopathic HES, reactive HES, or HEus.

A legend on the page’s upper side helps the user select the right element.

*HES (Hyperosinophilic Syndrome)* = Patient with >1500 Eos/mm3 with involvement of two or more organs, idiopathic in nature, associated with lymphoid or myeloid clonality, overlap disease, or reactive with another underlying condition (drugs, infections…).

*Lymphocytic variant* (L-HES): HES associated with clonal alterations to the immunophenotype on peripheral blood, TCR rearrangements

*Myeloid neoplastic disease* (M-HES): HES associated with gene rearrangements linked to haematological diseases, including PDGFR, FIP1L1, JAK2 or FGFR

*Familial HES*: cases characterised by familial clusters.

*Overlap forms*: ABPA, EGPA, chronic eosinophilic pneumonia, Gleich syndrome, eosinophilia-myalgia syndrome, Omenn syndrome, ALPS, IBD, Sarcoidosis, IgG4-related disease.

*Reactive forms*: forms of HES secondary to drug intake, infection, neoplasia or other conditions.

*Idiopathic forms*: forms of HES that have no known underlying cause. This diagnosis of exclusion will be made at the end of the complete diagnostic workup.

*HE-us hypereosinophilia of uncertain significance:* = >1500 eos/mm3 in asymptomatic patients without signs of eosinophil-mediated damage.

*Lymphocytic HES (L-HES)*

The user can select the abnormalities found in the lymphocytes’ subset, such as the population CD3+CD4+, CD3+CD4+CD7-, CD3+CD4-CD8-, CD3+CD4+IFNg+, CD3+CD4+CD8+, or others. Moreover, any TCR rearrangements and bone marrow biopsy results can be described if available.

*Myeloid HES (M-HES)*

If a patient is diagnosed with M-HES, the clinician can select the gene mutation found, e.g., PDGFR-alfa, PDGFR-beta, FIP1L1, or PCM1-JAK2. The user can choose the “other” field with a free text field if the mutation is not listed. Finally, the results can be described if a bone marrow biopsy is available.

*Familial HES*

In rare cases of familial HES, two free text fields can host the relatives affected by eosinophilic disorders and the performed gene tests.

*Overlap forms of HES*

Patients affected with conditions associated with HE but with an entirely different pathogenesis than HES are diagnosed with overlapping forms of HES. The user will select between ABPA, EGPA, Chronic Eosinophilic Pneumonia, S. Di Gleich, Eosinophilia-Myalgia Syndrome, Omenn/Iper-IgE Syndrome, ALPS, IBD, Sarcoidosis, or IgG4-related disease.

*Idiopathic HES*

For a patient with idiopathic HES, any more information can be added in a dedicated free text field.

*Reactive HES*

Patients with a diagnosis of reactive HES are further classified according to the underlying reactive condition, including drugs, malignancy, infections or others.

If the cause *Drug* is selected, the system will ask whether or not the criteria for a DRESS are met. Among *Malignancies*, the following sites can be chosen: breast, uterus, ovary, lung, stomach, colon, bladder, lymphoma, or other sites. Finally, any of the following infections can be selected: Helminths, Ectoparasites (scabies), Protozoa, Fungi, Viruses, or Others. If none of the listed causes is responsible for the reactive HES, the user can select the section “Others” with a free text field.

*HEus*

If no organ damage is detected and no underlying causes for HE are detected, the HEus diagnosis is selected.

After the section referred to AEC is completed, the user will proceed to the “Signs and symptoms” section.

### Clinical manifestations - signs and symptoms

In this section, the user can choose among the potential organs involved in HES. In the case of a patient with a single-organ HES, with a previously chosen diagnosis (i.e. eosinophilic esophagitis), the system reminds the involved organ and immediately presents the symptoms related to that organ involvement.

Otherwise, one or more organs should be selected: Esophagus, Stomach, Duodenum, Colon, Lung, Kidney and urinary tract, Skin and mucosae, Vascular and haematological systems, Muscular system, Heart, Peripheral Nervous System, Central Nervous System, ENT tract, Eye, Other, No organs.

- *Esophagus, Stomach, Duodenum or Colon:* If one or more gastrointestinal organs are selected, the user can choose one or more items among the following: Bolus, Dysphagia, Nausea and/or vomiting, Anorexia, Abdominal discomfort, Abdominal pain, Abdominal distension, Heartburn, Diarrhoea, Ascites, Dyspepsia, Other.
- *Lung:* If the lung is selected, the user can choose one or more items among the following: Dyspnoea, Productive cough, Non-productive cough, Wheezing, Asthma attack, Chest tightness, Haemoptysis, Haemophthisis, Pleural effusion, No symptoms, Other.
- *Kidney and urinary tract:* If the kidney or urinary tract is chosen, the user can select one or more items: Urinary incontinence or urgency, Oligo-anuria, Prostatic pain, Proteinuria, Bladder globe, Painful urination, No symptoms, Other.
- *Skin and mucosae:* If the “skin and mucosae” section is selected, the user can choose Dyschromia, Eczema or lichenification, Functional inability, Urticaria, Urticaria vasculitis, Painful swelling, Mucous ulcers, Purpura, Itching, Angioedema, Bullous lesions, Other.
- *Vascular and haematological systems:* If the kidney or urinary tract is chosen, the user can select one or more items: Deep venous thrombosis (DVT), Transitory Ischemic Attack (TIA), Superficial Phlebitis, Aneurysm, Arterial thrombosis, Carotid atheromasia, Stroke, Other.
- *Muscular system:* If the muscular system section is selected, the user can choose Muscle pain, Hyposthenia in upper limbs, Hyposthenia in lower limbs, Functional inability, or Other.
- *Heart:* If the Heart section is chosen, the user can select one or more items: Heart-pounding, Signs of heart failure, Dyspnoea, Dyspnoea, Arrhythmias, Chest pain, Pericardial effusion, Valvulopathy with recently detected heart murmur, No symptoms, Other.
- *Peripheral Nervous System (PNS):* The selection of PNS suggests one or more clinical manifestations, as follows: Ataxia, Muscle atrophy, Hyposthenia, Painful paresthesias, anapallesthesia, burning pain, Other.
- Moreover, the kind of Neuropathy should be clarified by selecting between sensory, motor or both. In the second step, peripheral motor neuropathy should be assessed by selecting Polyneuropathy, Multiple mononeuritis or Unknown. The exact location of the neuropathy can also be better explained in a free-text field.
- *Central Nervous System (CNS):* If the CNS section is chosen, the user can select one or more items: headache, vomiting, dizziness, stasis papilla, lethargy, visual changes, side signs, or other.
- *ENT tract:* If the ENT section is chosen, the user can select hearing loss, vertigo, mastoiditis, nasal obstruction, rhinorrhoea, frontal headache, pharyngitis, dysphonia, or other.
- *Eye:* If the eye is involved, the user can select: bulbodynia, photophobia, lacrimation, xerophthalmia, conjunctival hyperemia, scleromalacia, eyelid oedema, or other.
- *No organs:* If the user has previously chosen “HEus”, the platform automatically selects the “No organ involvement” button. If the user tries to choose organ involvement, a pop-up message appears, indicating that organ involvement does not fit the Heus definition.

Moreover, if the button “Other” is selected, the free-text field “Other symptom at onset” will appear every time. Finally, for every organ involvement, the user should indicate the period (month, year) of onset of the symptoms due to the potential asynchronous onset of clinical manifestations.

### Lab tests

The section concerning blood tests includes the following tests.

- *Routine tests:* complete blood count (WBC, absolute eosinophils count, Hb, Plts), ESR, CRP, serum creatinine levels, AST, ALT, GGT, total bilirubin, LDH, Fibrinogen, CPK.
- *Vitamins levels*: Vitamin B12, Ferritin, Transferrin.
- *Allergy tests*: total IgE, specific IgE for aspergillus fumigatus, recombinant IgE for aspergillus fumigatus (Asp f1, Asp f4, Asp f6) or other specific IgEs.
- *Immunology lab tests*: lymphocytes subset including any clonal subset expression or TCR rearrangement, serum IgG4 levels, basal serum tryptase, and ANCA (p-ANCA and c-ANCA).

- Finally, serum assessment of NT-proBNP and Troponin T/I can be stored to assess potential heart involvement.

### Instrumental tests

The fourth section of the platform allows the user to store data concerning the instrumental tests, confirming the eosinophilic involvement of target organs.

According to the organ involvement the users declared in the previous section, the system will ask about the medical exams required for HES or HEus diagnosis, as follows:

- *Gastrointestinal involvement:* if performed, upper or lower endoscopy with histological examination. A free-text field allows the user to describe the macroscopical aspects of the endoscopy, whereas the microscopical examination includes the number of eosinophilic in the examined samples.
- *Lung involvement:* the user can clarify whether or not the patient had a history of asthma, CRSwNP/CRSsNP, and whether or not a biopsy was performed. In case a biopsy is done, the user should add the location of the biopsy in a free-text field. A second step allows the user to insert the lung function test results (FEV1, FVC, FEV1/FVC, RV, TLC, DLCO) and nasal or bronchial FeNO. If a bronchial reversibility test was performed, the kind of bronchial response was asked (partial, complete, or none), and a question about eosinophils in the broncho-alveolar lavage was made. In addition, information about an allergy visit is asked, and skin prick test results are requested.
- *Kidney and urinary tract:* Information about the eosinophil count on tissue biopsy (bladder, prostate, kidney) is requested.
- *Skin and mucosae:* Information about the eosinophil count on tissue biopsy (skin, subcutaneous layer, oral mucosae) is requested.
- *Vascular and haematological systems:* the user can select what instrumental exam the patient underwent among Doppler-US of lower limbs, Doppler-US of supra-aortic trunks, Arteriography, CT angiography, MRI angiography or other. In any case, a free-text field will appear, allowing the user to describe the findings and the site of the imaging exam.
- *Muscular system:* Information about the eosinophil count on tissue biopsy in muscle is requested, as well as the electromyography results.
- *Heart:* in the case of a heart-US was made, information about atrial enlargement, ventricular dilatation, ventricular hypokinesia, left ventricular hypertrophy, pericardial effusion, and signs of ischaemia are requested. If an ECG was done, the following fields could be selected: unremarkable, Repolarisation alterations, Signs of ischaemia, atrioventricular block, bundle branch block, axial deviation, NSTEMI, STEMI, and others. Finally, if a cardiac biopsy is performed, the user can put its results.
- *Peripheral Nervous System (PNS) or Central Nervous System (CNS):* the system asks for information about the results of EMG, motor or sensorial evoked potentials, brain MRI, brain TC scan, and spinal fluid analysis.
- *ENT tract:* In the case of ENT involvement, this section requests the results of a sinus CT scan, nasal fibre-optic rhinoscopy, nasal cytology, nasal fluid, or nasal washing. Moreover, the user can add information in this section if the patient has already undergone FESS surgery. All this information can be added as a free-text field.
- *Eye:* a free-text field that hosts all the data concerning eye involvement.

In the case of *“No organs”* selected, the system double-checks that all exams have been made. It reminds us that the chest CT scan, upper and lower GI endoscopy, lung function tests, EMG/ENG, ECG, Echocardiogram, brain MRI, bone marrow biopsy and Abdominal ultrasound are mandatory.

### Therapy

In the “Therapy” section, the user can select one or more therapeutic approaches indicated in HES patients:

Corticosteroids: OCS, Oral Budesonide, CS topical nasal, CCS + nasal AntiH1 (e.g. fluticasone/azelastine, mometasone/olopatadine).

Inhaled asthma treatments: ICS, ICS/LABA, ICS/LABA/LAMA, LAMA, LTRA (e.g. Montelukast)

Biologics mAbs: Mepolizumab, Benralizumab, Omalizumab, Dupilumab, Tezepelumab

Haematological therapies: Hydroxyurea, IFN-alpha, Imatinib, Alemtuzumab

DMARDs: MTX, Cyclophosphamide, Cyclosporin, Tofacitinib, Upadacitinib, Baricitinib, Filgotinib, Azathioprine,

Other treatments: PPI, treatments for the underlying disorders (e.g. chemotherapy for tumours, anthelmintic), and others, or no therapies.

Among the non-pharmaceutical approaches, the exclusion diet is the only one included.

After any of the treatments above are selected, a time field will appear, including the starting and ending dates. The dose should also be added if the treatments have different dosages (e.g., mepolizumab 100 mg vs. 300 mg, or OCS).

### Follow-up and remission

The last section of the platform includes information on the follow-up duration (in months) and the achievement of remission either at one, three, twelve, eighteen, or twenty-four months or at the time of the questionnaire fulfilment. If a patient does not achieve remission, defined as having no flares in the last six months, a dedicated button should be pushed. Moreover, information about the patient’s age at the time of the remission achievement should be given.

### Anticipated results

Thanks to the platform’s combined prospective and retrospective data collection capabilities, we anticipate obtaining a comprehensive and dynamic real-world picture of patients affected by eosinophilic-related disorders across Italy. This dual design will allow us to capture both historical and current clinical trends, enabling the analysis of disease progression, treatment patterns, and patient outcomes over time. Importantly, the platform will also shed light on the heterogeneity of diagnostic and therapeutic approaches adopted by various Italian Centres of Excellence. Such insight will be crucial in harmonizing clinical practices, identifying unmet needs, and ultimately improving the management of these highly complex patient populations.

### Future perspectives

Future perspectives for Phleos are centered on its international expansion and integration into a broader research and clinical network. Initially developed and implemented across Italy, the platform is currently being translated into multiple languages, with English as the primary priority, to ensure accessibility and usability for a global audience. This multilingual adaptation represents the first step toward its dissemination across Europe and beyond. By fostering international collaboration, the platform aims to standardize data collection, enhance interoperability among centers, and support large-scale observational studies and clinical trials. Ultimately, this initiative will contribute to the development of shared diagnostic and therapeutic strategies, enabling a more comprehensive understanding and management of eosinophil-associated diseases worldwide.

## Discussion

HES currently represents a significant interest in clinical and translational research aimed at clarifying the underlying mechanisms and overall improving disease management (3).

A critical challenge remains the timely recognition of affected individuals and the prompt initiation of personalized therapies—an aspect that must be prioritized to reduce diagnostic delays and therapeutic inertia. The INHES platform was specifically developed to address these challenges, supporting clinicians and patients through a structured, data-driven approach to the complexity of HES. While multidisciplinary collaboration—including gastroenterologists, dermatologists, pulmonologists, cardiologists, and pathologists—is essential for comprehensive care, the pivotal role of allergists and clinical immunologists must be emphasized (6). These specialists possess the expertise required to interpret the protean manifestations of eosinophil-related disorders, integrate immunopathological insights, and guide the diagnostic and therapeutic process. Their central involvement is indispensable for ensuring accurate classification, optimal treatment selection, and coordinated care across multiple organ systems.

## Data Availability

The raw data supporting the conclusions of this article will be made available by the authors, without undue reservation.

## References

[1] Caminati M Brussino L Carlucci M Carlucci P Carpagnano LF Caruso C . Managing patients with hypereosinophilic syndrome: A statement from the italian society of allergy, asthma, and clinical immunology (SIAAIC). nCells. (2024) 13:1180. doi: 10.3390/cells13141180, PMID: 39056762 PMC11274683

[2] Caminati M Carpagnano LF Alberti C Amaddeo F Bixio R Caldart F . Multidisciplinary Group on rare dysimmune conditions with hyper-Eosinophilia (GEos). Idiopathic hypereosinophilic syndromes and rare dysimmune conditions associated with hyper-eosinophilia in practice: An innovative multidisciplinary approach. World Allergy Organ J. (2024) 17:100928. doi: 10.1016/j.waojou.2024.100928, PMID: 39156600 PMC11327453

[3] Wechsler ME Hellmich B Cid MC Jayne D Tian X Baylis L . Unmet needs and evidence gaps in hypereosinophilic syndrome and eosinophilic granulomatosis with polyangiitis. J Allergy Clin Immunol. (2023) 151:1415–28. doi: 10.1016/j.jaci.2023.03.011, PMID: 37086239

[4] Miravitlles M Nuvez A Torres-Duran M Casas-Maldonado F Rodriguez-Hermosa JL Lopez-Campos JL . The importance of reference centers and registries for rare diseases: the example of alpha-1 antitrypsin deficiency. COPD. (2020) 17:346–54. doi: 10.1080/15412555.2020.1795824, PMID: 32791925

[5] Stratton C Taylor A Konstantinidis M McNiven V Kannu P Gill P . Barriers and facilitators to designing, maintaining, and utilizing rare disease patient registries: a scoping review protocol. JBI Evid Synth. (2024). doi: 10.11124/JBIES-24-00091, PMID: 39428992

[6] Marra AM Rossi CM Piga MA Moroncini G Bilò MB . Eosinophil-associated diseases: the Allergist’s and Clinical Immunologist’s perspective. Eur Ann Allergy Clin Immunol. (2024) 56:195–209. doi: 10.23822/EurAnnACI.1764-1489.339, PMID: 38546414

